# Molecular and physiologic basis of quinoline drug resistance in *Plasmodium falciparum* malaria

**DOI:** 10.2217/fmb.09.15

**Published:** 2009-05-05

**Authors:** Paul D Roepe

**Affiliations:** 1 Department of Chemistry and Department of Biochemistry, Cellular & Molecular Biology, and Center for Infectious Disease, Georgetown University, Washington, DC 20057, USA. Tel.: +1 202 587 7300; Fax: +1 202 687 7186; roepep@georgetown.edu

**Keywords:** digestive vacuole, hemozoin, multidrug resistance, *Plasmodium falciparum* chloroquine resistance transporter

## Abstract

30 years before the discovery of the *pfcrt* gene, altered cellular drug accumulation in drug-resistant malarial parasites had been well documented. Heme released from catabolized hemoglobin was thought to be a key target for quinoline drugs, and additional modifications to quinoline drug structure in order to improve activity against chloroquine-resistant malaria were performed in a few laboratories. However, parasite cell culture methods were still in their infancy, assays for drug susceptibility were not well standardized, and the power of malarial genetics was decades away. The last 10 years have witnessed explosive progress in elucidation of the biochemistry of chloroquine resistance. This review briefly summarizes that progress, and discusses where additional work is needed.

Human malarias are caused by infection with one of five malarial (*Plasmodium*) parasites (*Plasmodium ovale*, *P. malariae*, *P. knowlesi*, *P. vivax* and *P. falciparum*), which occurs during a female Anopheles mosquito blood meal (*P. knowlesi* is zoonotic, the others pass human to human). *P. falciparum* infections account for 95% of malaria mortality worldwide, so historically most malaria research has focused on this species. However, there is also a critical need for basic research on the others, particularly *P. vivax*. In *P. falciparum* malaria, sporozoites injected during the blood meal migrate through the skin via multiple routes, localize to the liver within hours, invade hepatocytes and are then released into the blood after approximately 2 weeks as large merosomes containing 5–10,000 new haploid merozoites. These then rapidly invade red blood cells (RBCs) [1] and proceed through an amazing differentiation and feeding cycle that is 45–50 h long. An initial ‘ring’ differentiates into a single trophozoite, which then differentiates to 8–36 new merozoites. These are then released from lysed infected RBC (iRBC) and re-invade RBCs once again. The resulting increased parasitemia generates the well known symptoms of fever, anemia, and so on, which without treatment can ultimately progress to severe or cerebral malaria, coma and death. Sporadically, iRBCs commit to gametocyte differentiation via poorly understood pathways. Haploid gametocytes ingested by another mosquito feeding on an infected human, differentiate to male and female and combine in the mosquito to produce diploid stages that ultimately result in the injection of new sporozoites via mosquito saliva during another blood meal.

Most clinical manifestations of malaria are associated with the iRBC cycle. Not coincidentally then, nearly all known antimalarial drugs act against the iRBC. Understanding unique parasite biochemistry within the iRBC is essential since it is intricately linked to the molecular mechanisms of drug action and drug resistance for virtually all known antimalarial drugs. The past 10 years has shown that as these mechanisms are increasingly defined in molecular detail, this guides the rapid development of additional inexpensive antimalarials active against chloroquine (CQ) resistant (CQR) *P. falciparum*. Promising advances with partially protective vaccines notwithstanding, new antimalarial drugs will be direly needed for at least the next two decades, and probably longer. The above is just a glimpse at a very complex host–parasite biology that varies for different species and that protects multiple parasite stages from effective immune-system intervention in multiple ways. Childhood versus adult versus pregnancy-associated malarias differ, and common co-infections involving malarial parasites and other microbes pose additional therapeutic challenges. As a consequence, multiple vaccines will most likely be needed to eradicate malaria. Thus, it is imperative that equal emphasis be placed on inexpensive drug development and other approaches for controlling malaria.

This review briefly summarizes progress related to inexpensive antimalarial drug development on three fronts:

- ▪ Definition of quinoline antimalarial drug pharmacology
- ▪ Molecular genetics of CQ resistance in *P. falciparum*
- ▪ Altered biochemistry and physiology linked to the genetics of CQR *P. falciparum*

It is a comprehensive picture of all three that teaches us how to circumvent quinoline antimalarial drug resistance. To date, three of the best five antimalarials of all time have been quinolines, and recent work suggests that, with a comprehensive understanding, additional inexpensive, effective quinolines can be developed quickly.

## Mechanism of quinoline action

Rapid RBC growth and subsequent multiplex asexual division presents severe metabolic challenges. The parasite has adapted to these challenges by acquiring the ability to rapidly digest millimolar levels of hemoglobin (Hb) acquired from the RBC cytosol. Hb digestion via elegant internalization and proteolysis within a unique lysosomal-like organelle termed the digestive vacuole (DV) is essential for parasite survival. The toxic by product, heme, released upon Hb proteolysis, cannot be degraded by the parasite because it lacks the heme oxygenase pathway. Instead, the parasite is one of only a handful of organisms on the planet that detoxifies copious amounts of heme by crystallization to hemozoin (Hz), as described later. Thus, this pathway is a specific, biochemically unique drug target. Key molecular details of this pathway have been elucidated in recent years. In parallel, drug-resistance researchers are defining how drug-pathway interactions are ‘hijacked’ by the drug-resistant parasite via subtle alterations in DV biochemistry and physiology. In a desperate time, some optimism is on the horizon.

The most successful antimalarial drug to date is the 7-chloro-4-amino-quinoline, chloroquine, which targets the Hb digestion pathway. The mechanism is not completely understood [1–6] but a critical facet is the inhibition of ferriprotoporphyrin IX (FPIX) heme crystallization to Hz [5–10]. As early as 1964 [11] the likelihood of important CQ–FPIX interactions was realized, and the idea gained momentum via the work of Fitch [12]. The Hz crystal unit is a curious ‘head to tail’ FPIX dimer, wherein oxygens, coordinating adjacent irons in the two tetrapyrrole rings, are donated by FPIX propionic acid side chains [13]. Formation of this dimer within the DV competes with the formation of the better studied µ-oxo dimer. In simple aqueous solutions the µ-oxo dimer is favored over the head to tail dimer. Overall, equilibria between multiple chemical forms of FPIX within the DV is poorly understood. However, it is known that both FPIX dimerization and Hz crystallization are highly pH dependent, and that Hz formation is accelerated by lipids and is obligatory for parasite survival.

A number of drugs, including CQ, potently inhibit the formation of Hz *in vitro*. Provocatively, *in vitro* IC_50_ for Hz crystallization sometimes (but not always) correlates with antimalarial IC_50_ *in vivo* [14]. However, this does not mean that every quinoline drug acts on the same chemical form of FPIX or at the same pH optimum. The kinetics of Hz inhibition for different drugs against various CQ sensitive (CQS) or CQR strains of malaria are just beginning to be defined *in vivo*, because crystals form in an irregular shape and are exceedingly difficult to image quantitatively within the live parasite [15].

Different molecular models for how CQ inhibits Hz formation have been proposed, including the lysosomotrophic model wherein changes in DV pH caused by CQ diffusion titrate either Hb digestion, FPIX derivatization, Hz crystallization, or some combination. Alternatively, CQ might inhibit a heme/enzyme complex that forms as an intermediate. However, most evidence suggests that CQ and other antimalarials bind directly to one or more chemical forms (monomers, dimers) of FPIX [5,6,14,16–20]. These data suggest that quinolines directly inhibit Hz formation by sequestering one or more precursors of the crystal unit, or perhaps by interacting with growing faces of the Hz crystal [17]. It then follows that the CQ resistance mechanism involves the disruption of one or more of these CQ–FPIX interactions. Importantly, since FPIX is made by the host (not the parasite), the target cannot be mutated, and since its release is an inevitable consequence of essential Hb digestion its availability cannot be reduced. Thus two of the most common routes to drug resistance are eliminated (e.g., mutation or decreased expression of target). To become CQR the parasite must therefore either compartmentalize the drug in a different way, alter signal transduction related to death or modify the target in some biochemically unique way. Indeed, Bray *et al.* extrapolated CQ-binding data and determined that intracellular target affinity differs for CQS versus CQR parasites [9], so some biochemical transformation of FPIX for CQR versus CQS parasites seems probable. Another study demonstrated this directly *in vivo* [17]. Recently, we have been the first to determine the atomic-level structures of noncovalent CQ, quinine (QN), amodiaquine (AQ) and quinidine (QD)–µ-oxo dimer FPIX complexes [18,19] and these structures reveal a number of routes for altering FPIX–drug interactions. Remarkably, we have also discovered that when CQ and FPIX are added together under conditions that mimic that of the DV, a covalent (dative Fe–quinolinal N) FPIX–CQ complex is formed [20]. These discoveries enhance our understanding of how drugs inhibit Hz crystallization, and define important molecular features of CQR malaria. That is, noting the presence of at least two different CQ–FPIX complexes, two different FPIX dimers and the different pH dependencies for complex and dimer formation, we learn that there are several quite simple routes that the parasite can take to titrate multiple drug–heme target interactions. Indeed, slightly altered DV pH, volume, ionic or lipid composition for CQR versus CQS parasites [21,22] can easily alter the ratio of monomeric versus dimeric FPIX, the ratio of µ-oxo versus head to tail dimer, or ratios of multiple drug–heme complexes. Since even closely related quinoline antimalarials have different preferences and affinities for various chemical forms of FPIX, these data suggest that heme target-affinity differences for CQR parasites seen *in vivo* are easily explained via alterations in physiologic parameters (DV pH, volume, ionic and lipid composition and so on) known to exert enormous influence on Hz and drug–heme chemistry [23].

FPIX monomers and dimers have different affinities for lipids. Lipids and/or monoacyl glycerols found within the DV provide convenient catalysts or scaffolds for the formation of Hz [24,25], and different quinoline drugs have different affinities for both monomer and dimer in aqueous solution. Thus, understanding heme monomer–dimer equilibria is essential [14]. De Dios and colleagues have recently explored pH and drug perturbations of the monomer–µ-oxo dimer equilibrium in some detail, and have also measured how this is influenced by drugs and micelles of different charge that, to a first approximation, model various possible lipid phases [26,27]. In brief, remarkably, CQ is now known to favor binding to FPIX dimers whereas QN clearly favors the FPIX monomer [14]. In free solution, CQ actively promotes the formation of the µ-oxo dimer whereas QN stabilizes the monomer, both in a highly pH-dependent fashion [26]. Addition of a model lipid phase reveals pH-dependent lipid head group dependencies for monomer–dimer equilibria in the presence of quinoline drugs [27]. These are due to different aqueous versus lipid solubilities of monomeric versus dimeric FPIX, as well as different solubilities for these species complexed with quinoline drugs. This partitioning behavior is remarkably drug specific, even for related quinolines such as CQ compared with QN. Thus, even subtle changes in lipids or pH has profound effects on the efficiency of Hz production and has vastly different influences on the ability of even closely related drugs (e.g., CQ vs QN) to inhibit Hz formation. This makes sense since a nondrug-associated form of FPIX successfully partitioned into (or on) a lipid phase is likely to be essential for Hz production at the rate that has recently been directly measured *in vivo* [15], and because drugs, pH and lipid composition individually and synergistically perturb FPIX–lipid partitioning. Evidenced by reciprocal patterns of CQ resistance versus QN resistance in some strains (see later), these data further highlight that CQ and QN are really quite different drugs, and that the design of inexpensive second generation quinolines active against CQR malaria should independently target CQ and QN pharmacophore scaffolds.

While this progress in elucidating molecular details of quinoline pharmacology is critical, importantly, a recent report suggests that drug–heme interactions are likely not the only way in which the drug exerts a toxic effect – it is only one layer of a more complex collection of effects [28]. In this paper, cytotoxicity of CQ against different stages of iRBC parasites was quantified for both CQS and CQR parasites. Surprisingly, CQ was found to be nearly as toxic to rings and schizonts as to trophozoites. Although recent work by Elliot and colleagues shows that ring parasites do indeed begin degrading some small amounts of Hb, as proposed earlier, schizonts do not metabolize Hb nor produce Hz as trophozoites do [29,30]. The simple conclusion then is that different forms of free FPIX are not likely to be the only targets of quinoline drugs. Even more surprisingly, this study also demonstrated that *P. falciparum* CQ-resistance transporter (PfCRT) mutations confer approximately similar levels of CQ resistance to all three iRBC stages [28]. Since a fully formed DV membrane does not exist for ring stage parasites, this suggests that the PfCRT protein (see later section on PfCRT) is likely to have additional locations within the parasite, and more than one physiological function. The next few years of research will yield a more complex, but more complete molecular picture of quinoline antimalarial pharmacology.

## Emergence & spread of quinoline resistance

Although some new pharmacophores are in development, the three major classes of antimalarial drugs are the quinolines (e.g., CQ, mefloquine [MQ] and QN), the antifolates (pyrimethamine and cycloguanil), which both poison nucleotide biosynthesis by limiting essential folate cofactors, and the reactive endoperoxides (artemisin and derivatives) whose action is not well understood. Resistance to the first two classes is profound and widespread, and resistance to the third may be beginning to appear [31]. CQR parasites were first noticed 10–20 years after the introduction of the compound and CQ resistance was well documented in 1959 in Asia. Attempts to eliminate malaria (via insecticides and prophylactic use of CQ) possibly further promoted CQ resistance.

CQ resistance is both spreading and continuing to evolve via additional selective pressure. Continued evolution of CQ resistance, artemisin resistance, MQ resistance, QN resistance and so on, continuously produces new genotypes and pharmacologic phenotypes. When resistance to multiple drugs is seen, the strain is often called multidrug resistant (MDR). Molecularly speaking there are many MDR genotypes. Thus, a sometimes overlooked aspect of antimalarial drug discovery is that we are only in the midst of an ongoing phenomenon; what is relevant for one subspecies of CQR *P. falciparum* is not necessarily relevant for another. It is crucial to understand ‘subtypes’ of CQ resistance and to develop inexpensive therapy that is effective against multiple subtypes.

Several important subtypes can now be described pharmacologically, genetically and (to some extent) in terms of their physiology and biochemistry. As elucidated in several other reviews there are many parallels between drug resistance in tumor cells versus *P. falciparum* [6,8,23]. A key role for ATP-binding cassette (ABC) proteins was emphasized early on, however, this cannot explain all phenomena [8,23,32,33]. Many reports underscore an important role for ion transport in drug resistance phenomena [34–37] and it is now generally well accepted that dysregulation of ion transport is likely to be central to multidrug resistance in tumor cells, malarial parasites and some bacteria. This suggests that altered drug accumulation in MDR cells may be due, at least in part, to consequential alterations in pH gradients, volume and/or other biophysical parameters that control diffusion or accumulation of drugs; that signal transduction regulated by ion transport is a key player in resistance (this is certainly now clearly recognized for apoptotic signal transduction vs tumor multidrug resistance); and that the chemistry of drug–target interactions is manipulated via ion transport such that drug activity is then altered (e.g., the special case of highly pH-dependent FPIX–quinoline interactions described earlier).

Biochemical and physiological features shared among MDR tumor cells, malarial parasites and certain Gram-negative bacteria include altered intracellular accumulation of the drugs to which cells or micro-organisms are resistant, and that certain ‘chemo-modulators’ (e.g., the calcium channel blocker, verapamil [VPL]) can partially reverse the drug resistance. Although it is still not precisely understood how VPL functions as a chemoreversal agent, it is reasonable to expect that concepts from the study of MDR tumor cells will apply to MDR malarial parasites, and *vice versa*. Such was the hope when *pfmdr* genes were first cloned [38,39] and shown to be homologous to the *hsmdr1* gene, which is overexpressed in MDR tumor cells. It was initially thought that an ABC-protein drug pump for CQ must therefore exist in drug-resistant *P. falciparum*, similar to the drug pump proposed for tumor cells (human multidrug resistance protein 1 [HsMDR1], also known as Pgp) believed by many investigators to directly translocate vinblastine and many other anti-tumor drugs.

However, subsequent work showed that some drug-resistant *P. falciparum* strains do not encode mutated or overexpressed *pfmdr* [40,41], and that other genetic events must therefore be important. In parallel once again to tumor drug resistance, at the same time an increasing importance of the overexpression of other genes (e.g., ion transporter and mutant pro- and antiapoptotic genes) was recognized for MDR tumor cells [42,43]. Similar to *P. falciparum* multidrug resistance (PfMDR) protein versus malarial multidrug resistance (see later), the precise role of HsMDR1 protein in tumor multidrug resistance has been questioned for some time as pathology data have not correlated HsMDR1 overexpression with clinically relevant tumor multidrug resistance as strongly as was initially suspected [36,43,44].

It is now clear that there are multiple molecular layers to multidrug resistance pathways and that these continue to evolve. Elucidating the molecular function of proteins that appear to be membrane transporters and that are mutated in CQR malarial parasites (which are often also MDR) remains a critical area of study and is briefly summarized in the last section of this review.

## Initial genetic studies: cg2 versus Na^+^/H^+^ exchange; the wrong gene but the correct physiology

A genetic definition of CQ resistance began with the cloning of *pfcg2* [45], a technical tour de force essentially started years earlier via the successful creation of CQS × CQR cross progeny [46]. Correlation between specific *pfcg2* sequence and CQS or CQR status was found for all but one strain of *P. falciparum* examined. The one exception, strain Sudan 106/1, turned out to have a special utility in characterizing the true CQ resistance determinant (see later section on PfCRT). Prior to that work, Lanzer *et al.* concluded that mutated PfCG2 protein was a dysregulated Na^+^/H^+^ exchanger (NHE) that also pumped CQ out of the cell [47,48]. Wellems *et al.* questioned this because subcellular localization revealed PfCG2 in vesicle-like structures near the parasitophorous vacuolar space and the DV [49,50], not within the plasma membrane as envisioned by Lanzer *et al* [47]. Follow-up studies by Bray and Ward did not measure any Na^+^ dependency for CQ resistance, arguing against a strong role for NHE in influencing CQ transport or any other feature of CQ resistance [51]. Most recently, quantitative trait loci (QTL) analysis, availability of the *P. falciparum* genome and novel single-cell imaging in an extensive series of drug-resistant progeny suggested that altered NHE and cytosolic pH (pH_cyt_) are indeed related to resistance, but that the relevant ion exchanger is not PfCG2 (see later section on *P. falciparum* Na^+^/H^+^ exchanger [PfNHE]), and that resistance to QN is more influenced by these changes than CQ resistance [52].

Wellems and colleagues subsequently proved, via direct transfection experiments, that mutant PfCG2 did not confer CQ resistance [53]. Simultaneously, the Roepe group measured alkaline pH_cyt_ for some but not all CQR parasites, and early NHE data by that group [54] led to conclusions similar to those of Bray and Ward [51]. Thus, attention then focused on another gene found within the same 36 kbp fragment that harbored *pfcg2*, namely, *pfcrt* [55]. Results described in this and additional papers show that PfCRT protein is the ultimate determinant of CQ resistance in *P. falciparum* malaria, that it resides within the DV membrane, and is not directly involved in pH_cyt_ regulation or plasma membrane NHE (see later section on PfCRT) [56,57].

Along with the now recognized dominant role of PfCRT, *P. falciparum* MDR protein 1 (PfMDR1) is now thought to only modulate cross-resistance patterns in CQR parasites (see later). Meaning, this transporter modifies the rank order of resistance to other drugs such as MQ [58,59]. Other proteins, including PfNHE1, which is a true NHE, are now suspected to complement drug resistance even further [52,60]. This degree of genetic complexity involving at least three membrane transporter genes is daunting, but is to be expected. Early explanations for MDR phenomena in either tumor cells, bacteria or parasites were (in hindsight) a bit too optimistically reductionist.

## More genetics; the elusive role of PfMDR1

As mentioned, early studies of CQ resistance in *P. falciparum* showed resistance was associated with decreased drug accumulation that was reversed by the ion-channel blocker VPL [33,61]. Similar phenomena had been seen in MDR tumor cells. Thus, Wirth and colleagues screened *P. falciparum* for *hsmdr1* homologs and identified *pfmdr1* and *pfmdr2* [38]. While both encode proteins expressed in drug-sensitive *P. falciparum*, a MQR strain showed elevated *pfmdr1* [54]. MQ is chemically similar to CQ, so at the time resistance pathways were expected to overlap. In support of this, another group found *pfmdr1* to be upregulated in some CQR *P. falciparum* [39]. However, subsequent experiments showed that *pfmdr1* overexpression did not correlate with CQ resistance [62]. This was not surprising since Wellems *et al.* had earlier shown that CQ resistance did not segregate with the *pfmdr1* chromosome 5 locus in progeny from a CQS × CQR genetic cross [46]. Proposals arguing against a dominant role for HsMDR1 protein in tumor resistance were also being made during this period, as ion transporters were found to be alternatively expressed in some MDR tumor cells well before overexpression of HsMDR1 [42].

On the other hand, polymorphisms in *pfmdr1* were also associated with CQ resistance early on [63]. While CQS isolates had identical PfMDR1 sequences, there were five changes in CQR isolates. In strains K1 and ITG2, N86Y was the only change. CQR strain 7G8 had four changes; Y184F, S1034C, N1042D and D1246Y [63]. The Y184F mutation was postulated as not likely to be involved in CQ resistance since it was also found in CQS strains. Thus, the *pfmdr1* overexpression hypothesis was revised to suggest that CQR strains expressed mutant PfMDR1 but did not necessarily overexpress wild-type [63]. (Which *pfmdr* alleles are considered ‘mutant’ versus ‘wild-type’ is open to interpretation, wild-type is used here to refer to the allele found in the CQS strain HB3.) Interestingly, CQR K1 versus 7G8 polymorphisms appear to be geographically biased [64–66].

Subsequently, when MQR *P. falciparum* were selected to higher levels of MQ resistance, *pfmdr1* was found to be amplified [67]. Halofantrine resistance and QN resistance increased with increasing *pfmdr1* whereas AQ resistance did not [67]. However, when CQR strain K1 was selected against halofantrine it did not result in MQ resistance or amplification of *pfmdr1* [68]. In a more recent study, which used allelic exchange of *pfmdr* to probe these questions, incorporation of *pfmdr1* 7G8 polymorphisms into a CQS strain not previously exposed to a drug had no effect on CQ resistance, but incorporating wild-type *pfmdr1* into a CQR strain expressing mutant *pfmdr1* did decrease the level of resistance by half [58]. CQS strains expressing mutant *pfmdr1* alleles showed some mild QN resistance and altered sensitivity to MQ [58]. Variations on this theme have also been described by Fidock and colleagues [59]. Collectively, these data suggest that PfMDR1 effects are subtle and strain-specific. This brings us to our current understanding: it seems unlikely that mutations in *pfmdr1* confer CQ resistance in and of themselves [69], but they (or overexpression of certain isoforms) provide an important modulatory effect [70].

## Still more genetics: PfCRT

As previously mentioned, early on, Wellems *et al.* showed that *pfmdr1* was unlikely to directly cause CQ resistance since the relevant region of chromosome 5 did not segregate with the CQ resistance phenotype in genetic cross progeny [46]. A follow-up paper suggested that CQ resistance segregated with the *pfcg2* gene on chromosome 7 [45], but this paper also showed that one CQS strain (Sudan 106/1) carried CQ resistance-associated *pfcg2* yet was nonetheless CQS. The 36 kbp chromosome 7 locus harboring *pfcg2* that segregated with CQ resistance was thus re-examined and a previously unrecognized gene, now known as *pfcrt* was found [55]. Mutations in *pfcrt* are the central determinant of *P. falciparum* CQ resistance. The 13 exons span 3.1 kbp and encode a 424 amino acid, 48.6 kDa protein. Mutant *pfcrt* alleles found in CQR parasites contain a number of point mutations that confer amino-acid substitutions, with the pattern depending on the region of the globe from which the CQR parasite originates. In general CQR parasites from southeast Asia and Africa carry seven to eight point mutations, whereas South American CQR strains carry five [55]. Novel patterns continue to be discovered [65] and now at least 12 distinct CQ resistance-associated PfCRT isoforms have been described.

The pattern of mutations provides identification of the probable geographic origin of a CQR isolate. The number of mutations apparently required for CQ resistance explains two riddles, namely, why CQ resistance took so long to appear on a large scale and why it had been impossible to create CQR strains from CQS strains in the laboratory via drug selection. However, if one begins with Sudan 106/1, a strain that harbors all but one of the mutations required to complete a CQR-*pfcrt* allele, CQR strains can be rapidly created in the laboratory by CQ selection [57], and the final mutation required to complete the CQR-*pfcrt* allele [55] is found in these selected strains. In performing this experiment, Cooper *et al.* also found PfCRT substitutions that are not known to exist in the wild but that confer unusual and scientifically informative drug-resistance profiles [57].

Similar to PfMDR1, PfCRT is localized to the DV membrane and is a polytopic, integral membrane protein that is likely to perform some type of transport function [21,22,55,71–76]. Similar to the case for HsMDR1 most initial hypotheses for its function suggest either ion or drug transport or both, since, again, CQR parasites accumulate less antimalarial drug in a given time relative to CQS and quinolinal antimalarial drugs (like anti-tumor drugs), which are hydrophobic weak bases. In fact, CQ and related drugs are dibasic and the DV is now known to be quite acidic. So, fold concentration of CQ within the DV (where the heme-drug target is found) is dependent upon the square of the net pH gradient and would be as much as 10^5^–10^6^-fold by the predictions of weak base partitioning theory, depending on relative permeabilities of neutral, +1 and +2 CQ species [6]. Thus, even very subtle changes in DV pH would have very significant consequences. Progress on these and related questions is summarized later.

## Na^+^/H^+^ exchange may indeed be involved in quinoline drug resistance

As mentioned previously, one additional genetic event that tailors CQ resistance caused by PfCRT, may be the mutation and/or over expression of PfMDR1, but this cannot fully explain all MDR phenotypes. Another contributing event, recently identified by QTL analysis [60], involves one or more genes encoded by a segment of chromosome 13. This fragment is hypothesized to contain genes encoding for proteins with unclear homology to known proteins, however, it also encodes homologs to a V-type ATPase subunit and to the NHE protein family. By combining QTL analysis with improved pH_cyt_ measurements, we have recently shown that PfNHE dysregulation is likely to be linked to one route to increased QN resistance [52]. In addition, additive QTLs on chromosomes 5 and 7 were found as expected (the identified fragments contain *pfcrt* and *pfmdr1*) [60]. Pairwise effects were also detected between chromosome 13 and a chromosome 9 locus. The NHE homolog encoded within chromosome 13 was named PfNHE1. This protein is the second largest eukaryotic NHE yet identified (surpassed only by a NHE for the related apicomplexan *Toxoplasma gondii*), and has several unusual features. Bioinformatic analysis and cross reactivity with anti-TgNHE antibody [Chen D, Pleeter P, Roepe PD, Unpublished Data] show that PfNHE is localized to the plasma membrane. Polymorphisms that encode variable DNNND repeats in the predicted PfNHE protein sequence have been found in progeny of a CQS × CQR cross, as well as a range of field isolates and additional laboratory strains showing variable QN resistance [60].

Prior to the availability of the *P. falciparum* genome, Ginsburg *et al.* quite logically suggested a plasma-membrane NHE must exist to decrease cytosolic acid caused by anaerobic glycolysis [77]. As mentioned earlier, Lanzer *et al.* suggested that *pfcg2* found close to *pfcrt* on chromosome 7 encoded this NHE [48], but this was disputed, since PfCG2 is actually peri-vacuolar [50], not plasma membrane localized, and because the putative PfCG2–NHE homology was based on sequence analysis that did not account for very high AT content in malarial genes [49]. Regardless, the field has come full circle and the initial conclusions by Lanzer *et al.* were indeed partly correct (the physiology was correct, but not the genetic explanation). In our hands single-cell photometry (SCP) analysis of pH_cyt_ for intra-erythrocytic parasites under continuous physiologic perfusion indeed show elevated pH_cyt_ for some CQR parasites, but not all [54,55,57]. A corollary we suggested is that the relative size of the net cytosolic–DV pH gradient might be a more important parameter for CQ resistance versus QN resistance, rather than steady-state pH_cyt_ or digestive vacuolar pH values alone [54]. Overall, a range of pH phenomena and genetic changes consistent with changes in pH regulation are associated with QN resistance and CQ resistance. As described [52], we recently optimized localization of the pH probe BCECF exclusively to the parasite cytosol to avoid complexities in interpretation from earlier studies, and showed that elevated pH_cyt_ is well correlated with QN resistance and increased apparent PfNHE activity [60]. We now believe there are at least two physiological signatures for QN resistance that segregate with the two genetic descriptions, and that one is alkaline pH_cyt_ [52]. Technical details of PfNHE measurements have recently been questioned [78], but these issues have hopefully been clarified [79].

## Altered DV biochemistry & physiology

Genetic definition of CQ resistance and related phenomena is a major breakthrough, but to develop drugs and other therapies the altered biochemistry and physiology linked to that genetics must also be elucidated. As described in the first part of this review a chief drug target is FPIX in the DV, so studies of DV biochemistry are particularly important.

A central characteristic of DV biochemistry and physiology is the high pH gradient (acid inside) that the organelle has. Regulation of DV pH is not fully understood, but it includes a V-type H^+^ ATPase that hydrolyzes cytosolic ATP to pump H^+^ into the DV [80]. Interestingly, although conflicting data have been published [81] it is now generally accepted that changes in DV pH and volume are linked to CQ resistance caused by PfCRT [21,22,74,76]. These are further predicted to affect drug, lipid, metabolite and osmolyte traffic in and out of the DV. However, since the DV is a subcellular organelle for an intracellular parasite, precise quantification of these perturbations is quite challenging. It requires the development of novel, overlapping approaches, as described later.

Our group attempted the first DV pH measurements for living intra-erythrocytic parasites under physiologic perfusion using the pH probe acridine orange (AO) and novel SCP [71,82]. Our initial hypothesis was that, owing to the dibasic nature of CQ, even subtle increases in DV pH would significantly lower DV concentrations of CQ and thus cause CQ resistance. Therefore, relative to CQS, CQR parasites might show slightly elevated DV pH or different DV pH behavior upon addition of CQ [83], or perhaps both. We and others have actually found that mutant PfCRT in CQR parasites causes more acidic (lower) DV pH. The initial AO data in support of this conclusion generated controversy (as most new technologies tend to do), but a number of different, complementary methods from several labs were subsequently developed and strongly supported the initial AO conclusions [21,22,74,76, 79].

Nonetheless, lower DV pH for CQR parasites appeared paradoxical, because simplistically it is predicted to concentrate more drug within the DV by weak base effects. It is difficult to see how concentrating more drug at the site of action works to confer drug resistance, but as pointed out [6,82] in hindsight the physiology is obvious when examined alongside the chemistry of the principle CQ target, FPIX released from Hb. As mentioned, iRBC parasites detoxify FPIX by crystallization to Hz. In aqueous solution, FPIX dimerizes to a µ-oxo dimer. FPIX has two propionic acid side chains, thus µ-oxo FPIX is a tetraprotic amphipathic weak acid with four equivalent titratable groups, and a pKa near DV pH [6,82]. As pH is dropped even subtly (0.1–0.3 units), because the titration curve of FPIX dimer with four equivalent pKa is exceedingly steep (shown in [82]), even low levels of soluble dimer convert to insoluble (aggregated) dimer over a very narrow pH range (<0.5 units [82]). CQ and other drugs bind well to soluble FPIX but not to aggregates. In addition, acid aggregation of FPIX accelerates conversion to Hz (to which drugs also do not bind well). For these reasons, lowering DV pH by as little as 0.1 units is actually predicted to be a potent pathway to CQ resistance [6]. Ingeniously, CQR parasites titrate one drug target to lower levels, and also bias pH-dependent FPIX chemistry. That is, even though at DV pH FPIX monomer is likely to be more abundant than µ-oxo dimer [26], FPIX equilibria are pulled away from monomer by acid aggregation phenomena. As explained in the first section of this review, it is now known that this simple picture is a bit more complex, and also involves quinoline drug-specific effects (e.g., CQ vs QN) on monomer–dimer equilibria and drug–FPIX aqueous versus lipid partitioning [26,27]. The overall point is that ion dependent CQ resistance DV biochemistry drives a number of chemical conversions that will act to disrupt quinoline drug–FPIX interactions in very potent ways. However, importantly, lower DV pH caused by CQ resistance-associated mutant PfCRT that correlates with a VPL reversible CQR phenotype can only be one aspect of the CQ resistance mechanism and cannot explain all quinoline drug resistance. For example, evidence suggests lower DV pH may not be directly related to QN resistance, and might even be related to MQ hypersensitivity in some cases [56,57]. As described earlier, added effects of proteins encoded by other identified loci (PfMDR, PfNHE) may hold clues to this complex spectrum of multidrug resistance phenomena. In addition, better definition of the molecular origins and repercussions of this altered pH is required. How does it occur? Is PfCRT a H^+^-coupled metabolite transporter that when mutated to a CQR form becomes partially uncoupled? Does PfCRT interact with the DV H^+^ ATPase? Does PfCRT transport a counterion (i.e., Cl^-^) to shunt membrane potential in the presence of a high change in pH, such that CQ resistance mutations alter anion flux to indirectly cause a greater pH change? Do PfCRT CQ resistance mutations change substrate specificities for a facilitative diffusion transporter? These questions all have very different predicted consequences.

Some cell-based experiments have attempted to address these questions. Lanzer and colleagues successfully expressed PfCRT in oocytes and measured pH, membrane potential and certain ion conductances across the oocyte membrane [83]. They noted that oocytes expressing PfCRT exhibited an altered transmembrane pH gradient and membrane potential due to H^+^ leak and somewhat nonspecific cation conductance. They proposed that PfCRT activates endogenous oocyte ion transporters in some way. Multiple molecular models that explain these observations are possible, but overall the data further highlight a role for PfCRT in ion and/or osmolyte traffic.

Another study followed ion dependencies for DV pH and volume regulation by imaging these parameters for live parasites under perfusion with a medium of altered salt composition [22]. Importantly, CQR parasites showed increased DV volume relative to CQS parasites, suggesting that pH and volume regulation are linked for the organelle, as is found for other acidic vesicles or lysosomes. However, fast transient changes in Cl^-^ gradients across the DV membrane did not lead to rapid changes in the DV transmembrane pH gradient, indicating no direct coupling of Cl^-^ and H^+^ transport. On the other hand, fast transient changes in DV Cl^-^ gradients were found to strongly influence DV volume. These effects were strongly CQ- and VPL-dependent and differed dramatically for CQS versus CQR parasites. The overall conclusion was that PfCRT mediates transport of an important DV osmolyte (presumably peptides, di-peptides or amino acids released from Hb digestion) and that this transport is altered for CQR parasites.

Taken together, the bulk of the evidence suggests that altered DV pH for CQR parasites is an indirect consequence of altered osmolyte traffic promoted by PfCRT mutation. This might be an explanation for why parasites treated in different ways (resulting in various levels of relevant osmolytes produced by a finely tuned metabolism) could perhaps show different DV pH in some studies [81,84]. If altered osmolyte traffic that then indirectly perturbs normal pH regulation is the explanation for altered DV physiology linked to PfCRT mutations, it begs the obvious question: what is the normal physiological function of PfCRT? An obvious attractive possibility suggested early on is that PfCRT might transport products of Hb digestion (peptides, dipeptides and/or free amino acids) [73,22], since these are among the most important DV osmolytes, since products of Hb digestion are unique osmolytes and PfCRT is a unique transporter with a unique sequence, and because when CQ, QN and other drugs are superimposed upon amino acid structures (e.g., CQ vs the cationic amino acid lysine), many interesting similarities can be noted. For example, the superposition (overlay) of a dipeptide N-terminal lysine (a common Hb amino acid) and CQ shows similar charge and a similar carbon chain flanked by nitrogen atoms. As another example, an OH group is bound to one chiral center of QN that is two s bonds away from the positively charged quinuclidine nitrogen, and a near identical spatial arrangement of stereochemically sensitive atoms is also found for one isomer of serine. Interestingly, QN and QD differ in their stereochemistry at this center yet show different PfCRT-isoform dependent pharmacology [57]. Perhaps not coincidentally then, if PfCRT did transport peptides or amino acids, substrate recognition would also be stereo-selective since only l-amino acids are found in Hb.

## Molecular analysis of mutated transporter function

Ultimately, genetics and cell physiology can only take us so far, and cannot fully resolve speculation regarding substrates of PfCRT or define the thermodynamics and kinetics of any protein-mediated transport. Elucidating the remaining questions will require detailed molecular studies with PfCRT, PfMDR1, PfNHE and perhaps other proteins yet to be described. However, the *P. falciparum* genome is anomalously AT rich, and some genes (including *pfcrt*) can have significant stretches of AT content that are 80% or more. These do not translate well in convenient heterologous systems such as bacteria or yeast. In fact, in the case of *pfcrt* and *pfmdr1* they do not translate at all. That is unfortunate, since techniques and strategies for defining transporter function have been elegantly laid out in bacteria and yeast model systems for the past four decades. Proteoliposomes, kabackosomes, Goffeau membranes, and Menendez phaseseparated yeast vesicle preparations would all prove incredibly useful for defining the molecular mechanism of CQ resistance, if only *pfcrt* cDNA could be expressed in either bacteria or yeast.

Hanbang Zhang working the Roepe laboratory succeeded in a brute force resolution to this dilemma. Noting the success of earlier gene design that allowed MSP-1 expression in *Escherichia coli* [85], Zhang back-translated the PfCRT amino-acid sequence using preferred yeast codons, then designed a set of overlapping 40-mer oligonucleotides that encoded both strands of the theoretically optimized sequence, and constructed an entirely synthetic gene via nested PCR methods. This enabled the expression of high levels of PfCRT protein in either *Saccharomyces cerevisiae* or *Pichia pastoris*, in either a constituitive or inducible fashion, respectively [72]. Analysis of ion transport for plasma membrane inside-out vesicles produced from these strains provided further evidence that PfCRT protein plays a role in ion transport [72], and purified membranes along with equilibrium binding studies, using 3H–CQ, provided the first direct evidence that PfCRT protein indeed binds CQ [73]. The latter is a central prediction for nearly all molecular hypotheses for PfCRT. Although not often cited, the same paper also provided the first direct molecular evidence that PfCRT likely transports CQ, via flow dialysis experiments using inside-out yeast plasma membrane vesicle with or without PfCRT protein [73]. The simplest model consistent with these results is that CQ transport by PfCRT is facilitative downhill diffusion at a rather low turnover. Different thermodynamic models for CQ transport (e.g., PfCRT as a CQ exchanger or active transporter) have also subsequently been proposed based on different approaches that measure 3H–CQ flux for live parasites [86,87]. Distinction between these models will require additional direct transport experiments at much higher kinetic resolution.

At the time, *pfcrt* was (to our knowledge) the largest synthetic gene ever constructed by overlapping PCR, nonetheless, success enticed others to attempt the reconstruction of a yeast-optimized *pfmdr1* gene, which is over three times the size of *pfcrt*. After numerous challenges were overcome, this too was achieved and unusual ATPase activity of PfMDR1 was rather quickly quantified via plate-based phosphate release assays [88]. Relative to other ABC transporters involved in drug-resistance phenomena, PfMDR1 has unusually high K_m_ and V_max_ for ATP hydrolysis. Also, interestingly, antimalarial drugs only mildly stimulated PfMDR1 ATPase activity, consistent with the notion that PfMDR1 exerts only mild affects on antimalarial drug resistance. With methods for *in vitro* analysis of PfMDR1 in hand, a follow-up study quickly defined which amino acid substitutions in the curious 7G8 PfMDR1 isoform confer its unusual and quite specific ATPase activity [89]. The S1034C mutation in 7G8 PfMDR1 is now known to abolish antimalarial drug-stimulated PfMDR1 ATPase activity. Although this molecular-level work is only just beginning, it has already significantly refined proposals for the molecular mechanisms of CQ resistance and antimalarial multidrug resistance.

Direct demonstration of CQ binding to PfCRT was a very important result, but equilibrium binding studies with radio-labeled CQ obviously do not allow for definition of the drug binding site. To answer this question, the Roepe and Wolf laboratories designed and synthesized various CQ photoaffinity probes. One particularly novel probe, haboring both per-fluoro-azido and biotin tags via flexible linkers attached to the aliphatic terminus of CQ, proved to be enormously useful. It has recently led to the identification of the CQ binding site in HB3 isoform PfCRT, and has also quickly quantified relative affinities of other drugs (QN, VPL and so on) versus various PfCRT isoforms [90]. An obvious extension of this work would be the definition of CQ binding-site differences (if any) for other PfCRT isoforms, and perhaps similar studies with PfMDR1.

The chemistry developed for construction of these photoaffinity probes leads to a rather straightforward design and synthesis of fluorescent CQ derivatives. Some dansyl–CQ derivatives actually turn out to be more active against CQR strains of *P. falciparum* than CQ, and are useful for imaging subcellular localization of CQ [91]. A more convenient probe that can be followed with more routinely available lasers and fluorescent microscopes, NBD–CQ, has also recently been developed [92]. Combined with vesicles and proteoliposomes as described for drug binding studies [73,90] this probe is currently allowing us to define putative CQ transport by PfCRT in very detailed molecular and thermodynamic terms.

## Conclusion

In principle, genetics, physiology and biochemistry related to altered FPIX chemistry and membrane transport now defines CQ resistance. It is my suspicion that permutations of the above will ultimately be shown to define resistance to all other quinolines (QN, AQ, MQ and so on), to acridines, xanthones and reactive endoperoxides, but probably not to antifolates, for which resistance involves other distinct pathways [93]. That is, at least three genes (*pfcrt*, *pfmdr1* and *pfnhe*) have been identified that, in various allelic combinations, confer what we now understand to be a spectrum of antimalarial multidrug resistance phenomena. The molecular function of at least two of these is becoming clearer via a combination of new chemical biology, artificial gene construction, and tried and trusted membrane biochemistry techniques. The physiologic and pharmacologic consequences of their function is at least qualitatively defined, and although there is still much more to do at the molecular level, the field has come a very long way in the 9 years since the identification of PfCRT. We can be hopeful that, based on this information, new quinolines effective against CQR malaria will prove increasingly easier to design and synthesize. In fact, the author suggests that this hope is even now being realized [91,94,95]. This work complements continued uses and combination therapies involving other quinoline derivatives such as pyronaridine (an aza-acridine) [96], piperaquine [97] and isoquine [98] as well as other heme-binding pharmacophores such as the very exciting xanthones pioneered by Riscoe and colleagues [99]. History repeatedly teaches us that even with the emergence of CQ resistance, inexpensive quinolines and related compounds can still be effective.

In summary, the PfCRT protein binds CQ as is predicted from several models for its function. Azido-biotin–CQ now clearly defines that binding site and distinguishes drug binding preferences for various PfCRT isoforms [90]. CQ resistance-associated mutant PfCRT shows altered CQ binding, promotes altered diffusion of CQ and perturbs osmolyte equilibrium within the DV, which then alters DV pH and volume regulation.

The latter influences FPIX chemistry and multiple FPIX–drug interactions in critical ways. Taken together, a more complex, but clearer molecular model for the mechanism of trophozoite CQ resistance is now possible [Paguio M, Cabrera M, Roepe PD, Submitted]. However, importantly, we should not neglect that trophozoite/DV-specific models for CQ resistance are clearly only one part of the story. Other effects of CQ and other layers of CQ resistance need to be elucidated [28]. Other aspects of CQ pharmacology must be present at the ring and schizont stages of *P. falciparum* development. Additional physiological functions for PfCRT at the ring and schizont stages must also exist and when altered by mutation also cause CQ resistance for these stages. A key question is how are these physiologic functions related to putative ion or osmolyte transport? For ring stages, based on the recent work of Elliot *et al.* it seems possible, at least in theory, that controlling Hz chemistry is again, at least, part of the explanation [29]. But for schizonts, the answer must be different. Perhaps mutant PfMDR1 and PfNHE further modulate drug resistance in the presence of mutant PfCRT for these stages as well. Their molecular level function can now also be studied using ‘yeast-optimized’ recombinant proteins and novel drug probes as described for PfCRT, so additional rapid progress is on the horizon.

## Future perspective

It is true that great strides have been made in understanding antimalarial drug resistance at a molecular level, and it is also true that this information is now turning out to be of great value in developing new, inexpensive, second-line antimalarials. ‘Molecular medicine’, well developed for western cancer and cardiology clinics, has (in theory) finally come to the malaria clinic – genes that dictate how the disease might respond to therapy have now been identified, and additional molecular tools developed with that knowledge are leading to new clinical successes. The function of proteins encoded by these genes is becoming understood. High-throughput screening methods such as the SybrGreen assay have led to fast, inexpensive ways to screen for novel antimalarials active against CQR malaria, at least at the pre-clinical stage. Multiple plasmodial genomes have become available, giving us unique insight into comparative genomics and many new drug targets to explore. New antimalarial drug leads are indeed rapidly appearing.

Yet, the expense and often difficult logistics of Phase II and III drug trials still prevent full implementation of these advances. The entire world yearns for vaccine development, but optimistically that is still many years in the future. In the meantime, inexpensive drugs are still desperately needed. In 5–10 years, we hope that additional emphasis will have been placed by the entire global community on the rapid development and deployment of inexpensive antimalarial drugs. More funding and effort directed towards a multi-tiered, balanced approach to malaria treatment and prevention is very much needed. For over 60 years, inexpensive antimalarial drugs have saved the lives of many millions of malaria victims. In theory, there is no scientific roadblock that cannot be overcome such that this continues to be the case well into the future.

## Author note

It has proven quite difficult to either increase or decrease expression of PfNHE protein to further test how PfNHE may be linked to QN resistance, but a new paper reports decreased PfNHE levels for three parasite strains (GC03, 1 BB5, 3 BA6) by stable transfection with a truncated 3´-untranslated region [100]. The transfectants from these experiments do not show statistically significant changes in resting (steady state) pH_cyt_ as would be expected based on previous work [52], but they do show small increases in QN sensitivity consistent with PfNHE protein being involved in QN resistance as proposed [60,52]. Unfortunately, in [100] only strains with low levels of QN resistance were amenable to transfection, whereas decreased PfNHE expression for strains with high levels of QN resistance would have been particularly informative. Also, the authors of [100] were unable to consistently clamp parasite pH_cyt_ to acid values in the absence of Na^+^, formally leaving open the question of whether decreased levels of PfNHE do indeed (as obviously expected) lead to a lower rate of Na^+^/H^+^ exchange. More work is needed with these and other transfectants, and better methods for analyzing Na^+^/H^+^ exchange for the intracellular parasite would clearly be helpful. Regardless, it is essential to recognize that effects on H^+^ transport and steady state pH due to lowering PfNHE expression [100] are certainly not expected to be analogous to the effects predicted from analysis of multiple pairwise loci influences on PfNHE activity [52]. Clearly, levels of QN resistance span a wide range, and PfNHE effects on QN resistance are complex, require multiple loci [52] and, as reported earlier, do not necessarily segregate exclusively with elevated pH_cyt_ [52]. Overall, as emphasized throughout this review, we now realize that multiple genetic alterations confer the molecular and physiologic basis of quinoline resistance patterns, and so it is to be expected that resistance phenotypes will involve multiple overlapping pathways.

**Executive summary**

- ▪ Over the past 9 years, a clear picture of the genetic alterations that accompany evolution of chloroquine (CQ) resistance in *Plasmodium falciparum* malaria has become available. *P. falciparum* CQ-resistance transporter (PfCRT) mutations must be present and these confer a tenfold decrease in CQ-mediated growth inhibition and toxicity. Mutations and/or elevated expression of *P. falciparum* multidrug-resistant protein 1 (PfMDR1), perhaps along with mutations in *P. falciparum* Na^+^/H^+^ exchanger, further tailor quinoline drug resistance, and perhaps resistance to other classes of compounds that also target the parasite digestive vacuole.

- ▪ To understand the physiological repercussions of these genetic alterations, additional cell culture and computerized microscopy methods have been developed. These are now rapidly providing new information on a number of key cell biological questions, including organellar biogenesis, CQS versus CQR parasite fitness, and metabolism.

- ▪ To uncover the molecular details behind how mutant proteins link to resistance phenomena function, heterologous expression, purification and reconstitution of at least two of them (PfCRT and PfMDR1) has recently been accomplished. Combined with new ‘chemical biology’ approaches, the field is now poised to fully describe the molecular mechanism(s) of CQ resistance.

- ▪ Taken together, these advances and others are accelerating development of new second-line antimalarial drugs active against CQR malaria.

## References

[1] Geary TG, Jensen JB, Ginsburg H: Uptake of (3H)chloroquine by drug sensitive and resistant strains of the human malaria parasite *Plasmodium falciparum* *Biochem. Pharmacol.* 35 3805–3812 (1986). 3535803 10.1016/0006-2952(86)90668-4

[2] Bray PG, Hawley SR, Mungthin M, Ward SA: Physicochemical properties correlated with drug resistance and the reversal of drug resistance in *Plasmodium falciparum* *Mol. Pharmacol.* 50 1559–1566 (1996). 8967978

[3] O’Neill PM, Bray PG, Hawley SR, Ward SA, Park BK: 4-aminoquinolines-past, present and future: a chemical perspective. *Pharmacol. Ther.* 77 29–58 (1998). 9500158 10.1016/s0163-7258(97)00084-3

[4] Krogstad DJ, Schlesinger PH: The basis of antimalarial action: non-weak base effects of chloroquine on acid vesicle pH. *J. Trop. Med. Hygiene* 36 213–220 (1987). 10.4269/ajtmh.1987.36.213 2435182

[5] Egan TJ: Interactions of quinoline antimalarials with hematin in solution. *J. Inorg. Biochem.* 100(5–6) 916–926 (2006). 16384600 10.1016/j.jinorgbio.2005.11.005

[6] Ursos LMB, Roepe PD: Recent progress in elucidating the molecular mechanism of resistance to the antimalarial drug chloroquine. *Med. Res. Rev.* 22(5) 465–491 (2002). 12210555 10.1002/med.10016

[7] Scholl PF, Tripathi AK, Sullivan DJ: Bioavailable iron and heme metabolism in *Plasmodium falciparum* *Curr. Top. Microbiol. Immunol.* 295 293–324 (2005). 16265896 10.1007/3-540-29088-5_12

[8] Bray PG, Ward SA: A comparison of the phenomenology and genetics of multidrug resistance in cancer cells and quinoline resistance in *Plasmodium falciparum* *Pharmacol. Ther.* 77 1–28 (1998). 9500157 10.1016/s0163-7258(97)00083-1

[9] Bray PG, Mungthin M, Ridley RG, Ward SA: Access to hematin: the basis of chloroquine resistance. *Mol. Pharmacol.* 54 170–179 (1998). 9658203 10.1124/mol.54.1.170

[10] Chong CR, Sullivan DJ Jr: Inhibition of heme crystal growth by antimalarials and other compounds: implications for drug discovery. *Biochem. Pharmacol.* 66(11) 2201–2212 (2003). 14609745 10.1016/j.bcp.2003.08.009

[11] Cohen SN, Phifer KO, Yielding KL: Complex formation between chloroquine and ferrihaemic acid *in vitro*, and its effect on the antimalarial action of chloroquine. *Nature* 202 805–806 (1964). 14187629 10.1038/202805a0

[12] Fitch CD: Chloroquine resistance in malaria: a deficiency of chloroquine binding. *Proc. Natl Acad. Sci. USA* 64(4) 1181–1187 (1969). 5271747 10.1073/pnas.64.4.1181 PMC223266

[13] Pagola S, Stephens PW, Bohle DS, Kosar AD, Madsen SK: The structure of malaria pigment b-haematin. *Nature* 404(6775) 307–310 (2000). 10749217 10.1038/35005132

[14] Dorn A, Vippagunta SR, Matile H, Jaquet C, Vennerstrom JL, Ridley RG: An assessment of drug–haematin polymerisation by quinoline antimalarials. *Biochem. Pharmacol.* 55 727–736 (1998). 9586944 10.1016/s0006-2952(97)00510-8

[15] Gligorijevic B, McAllister R, Urbach JS, Roepe PD: Spinning disk confocal microscopy of live, intraerythrocytic malarial parasites. 1. Quantification of hemozoin development for drug sensitive versus resistant malaria. *Biochemistry* 45(41) 12400–12410 (2006). 17029396 10.1021/bi061033f

[16] Dorn A, Stoffel R, Matile H, Bubendorf A, Ridley RG: Malarial hemazoin/ß-hematin supports heme polymerization in the absence of protein. *Nature* 374 269–271 (1995). 7885447 10.1038/374269a0

[17] Sullivan DJ Jr, Gluzman IY, Russell DG, GoldbergDE: On the molecular mechanism of chloroquine’s antimalarial action. *Proc. Natl Acad. Sci. USA* 93 11865–11870 (1996). 8876229 10.1073/pnas.93.21.11865 PMC38150

[18] Leed A, DuBay K, Ursos LMB, Sears D, de Dios AC, Roepe PD: Solution structures of antimalarial drug/heme complexes. *Biochemistry* 41 10245–10255 (2002). 12162739 10.1021/bi020195i

[19] de Dios AC, Casabianca LB, Kosar A, Roepe PD: Structure of the amodiaquine–FPIX µ oxo dimer solution complex at atomic resolution. *Inorg. Chem.* 43(25) 8078–8084 (2004). 15578847 10.1021/ic0489948

[20] de Dios AC, Tycko R, Ursos LMB, Roepe PD: NMR Studies of chloroquine–ferriprotoporphyrin IX complex. *J. Phys. Chem. A* 107 5821–5825 (2003).

[21] Bennett TN, Kosar AD, Ursos LMB *et al.*: Acidic digestive vacuolar pH of drug resistant *Plasmodium falciparum* *Mol. Biochem. Parasitol.* 133 99–114 (2004). 14668017 10.1016/j.molbiopara.2003.09.008

[22] Gligorijevic B, Bennett T, McAllister R, Urbach JS, Roepe PD: Spinning disk confocal microscopy of live, intraerythrocytic malarial parasites. 2. Altered vacuolar volume regulation in drug resistant malaria. *Biochemistry* 45(41) 12411–12423 (2006). 17029397 10.1021/bi0610348

[23] Roepe PD, Martiney JA: Are ion exchange processes central to understanding drug resistance phenomena? *Trends Pharmacol. Sci.* 20 62–65 (1998). 10.1016/s0165-6147(98)01282-6 10101966

[24] Pisciotta JM, Coppens I, Tripathi AK *et al.*: The role of neutral lipid nanospheres in *Plasmodium falciparum* haem crystallization. *Biochem. J.* 402(1) 197–204 (2007). 17044814 10.1042/BJ20060986 PMC1783988

[25] Jackson KE, Klonis N, Ferguson DJ, Adisa A, Dogovski C, Tilley L: Food vacuole-associated lipid bodies and heterogeneous lipid environments in the malaria parasite, *Plasmodium falciparum* *Mol. Microbiol.* 54(1) 109–122 (2004). 15458409 10.1111/j.1365-2958.2004.04284.x

[26] Casabianca LB, An D, Natarajan JK *et al.*: Quinine and chloroquine differentially perturb heme monomer–dimer equilibrium. *Inorg. Chem.* 47(13) 6077–6081 (2008). 18533646 10.1021/ic800440d PMC2584347

[27] Casabianca LB, Kallgren JB, Natarajan JK *et al.*: Antimalarial drugs and heme in detergent micelles: an NMR study. *J. Inorg. Biochem.* (2009) (Epub ahead of print). 10.1016/j.jinorgbio.2009.01.013 PMC2670948 19223262

[28] Gligorijevic B, Purdy K, Elliott DA, Cooper RA, Roepe PD: Stage independent chloroquine resistance and chloroquine toxicity revealed via spinning disk confocal microscopy. *Mol. Biochem. Parasitol.* 159(1) 7–23 (2008). 18281110 10.1016/j.molbiopara.2007.12.014 PMC2440633

[29] Elliott DA, McIntosh MT, Hosgood HD *et al.*: Four distinct pathways of hemoglobin uptake in the malaria parasite *Plasmodium falciparum* *Proc. Natl Acad. Sci. USA* 105(7) 2463–2468 (2008). 18263733 10.1073/pnas.0711067105 PMC2268159

[30] Francis SE, Sullivan DJ Jr, Goldberg DE: Hemoglobin metabolism in the malaria parasite *Plasmodium falciparum* *Annu. Rev. Microbiol.* 51 97–123 (1997). 9343345 10.1146/annurev.micro.51.1.97

[31] Noedl H, Se Y, Schaecher K, Smith BL, Socheat D, Fukuda MM: Artemisinin Resistance in Cambodia 1 (ARC1) Study Consortium. Evidence of artemisinin-resistant malaria in western Cambodia. *N. Engl. J. Med.* 359(24) 2619–2620 (2008). 19064625 10.1056/NEJMc0805011

[32] Bray PG, Hawley SR, Ward SA: 4-aminoquinoline resistance of *Plasmodium falciparum*: insights from the study of amodiaquine uptake. *Mol. Pharmacol.* 50 1551–1558 (1996). 8967977

[33] Martiney JA, Cerami A, Slater AFG: Verapamil reversal of chloroquine resistance in the malaria parasite *Plasmodium falciparum* is specific for resistant parasites and independent of the weak base effect. *J. Biol. Chem.* 270 22393–22398 (1995). 7673225 10.1074/jbc.270.38.22393

[34] Vargas RC, Garcia-Salcedo R, Tenreiro S *et al.*: *Saccharomyces cerevisiae* multidrug resistance transporter Qdr2 is implicated in potassium uptake, providing a physiological advantage to quinidine-stressed cells. *Eukaryot. Cell* 6(2) 134–142 (2007). 17189489 10.1128/EC.00290-06 PMC1797947

[35] Dzekunov S, Ursos L, Roepe PD: Digestive vacuolar pH of intact intraerythrocytic *P. falciparum* either sensitive or resistant to chloroquine. *Mol. Biochem. Parasitol.* 110 107–124 (2000). 10989149 10.1016/s0166-6851(00)00261-9

[36] Hoffman MM, Roepe PD: Analysis of ion transport perturbations caused by hu MDR 1 protein overexpression. *Biochemistry* 36 11153–11168 (1997). 9287158 10.1021/bi970530g

[37] Cheng J, Guffanti AA, Krulwich TA: The chromosomal tetracycline resistance locus of *Bacillus subtilis* encodes a Na^+^/H^+^ antiporter that is physiologically important at elevated pH. *J. Biol. Chem.* 269 27365–27371 (1994). 7961647

[38] Wilson CM, Serrano AE, Wasley A, Bogenschutz MP, Shankar AH, Wirth DF: Amplification of a gene related to mammalian *mdr* genes in drug-resistant *Plasmodium falciparum* *Science* 244 1184–1186 (1989). 2658061 10.1126/science.2658061

[39] Foote SJ, Thompson JK, Cowman AF, Kemp DJ: Amplification of the mulidrug resistance gene in some chloroquine-resistant isolates of *P. falciparum* *Cell* 57 921–930 (1989). 2701941 10.1016/0092-8674(89)90330-9

[40] Wellems TE, Panton LJ, Gluzman IY *et al.*: Chloroquine resistance not linked to *mdr*-like genes in a *Plasmodium falciparum* cross. *Nature* 345 253–255 (1990). 1970614 10.1038/345253a0

[41] Barnes DA, Foote SJ, Galatis D, Kemp DJ, Cowman AF: Selection for high-level chloroquine resistance results in deamplification of the *pfmdr1* gene and increased sensitivity to mefloquine in *Plasmodium falciparum* *EMBO J.* 11 3067–3075 (1992). 1353446 10.1002/j.1460-2075.1992.tb05378.x PMC556790

[42] Roepe PD, Wei L-Y, Cruz J, Carlson D: Lower electrical membrane potential and altered pHi homeostasis in multidrug resistant cells: further characterization of a series of MDR cell lines expressing different levels of P-glycoprotein. *Biochemistry* 32 11042–11056 (1993). 8105888 10.1021/bi00092a014

[43] Lowe SW, Ruley HE, Jacks T, Housman DE: p53-dependent apoptosis modulates the cytotoxicity of anticancer agents. *Cell* 74(6) 957–967 (1993). 8402885 10.1016/0092-8674(93)90719-7

[44] den Boer ML, Pieters R, Kazemier KM *et al.*: Relationship between major vault protein/lung resistance protein, multidrug resistance-associated protein, P-glycoprotein expression, and drug resistance in childhood leukemia. *Blood* 91(6) 2092–2098 (1998). 9490695

[45] Su X, Kirkman LA, Fujioka H, Wellems TE: Complex polymorphisms in an 330 kDa protein are linked to chloroquine-resistant *P. falciparum* in Southeast Asia and Africa. *Cell* 91 593–603 (1997). 9393853 10.1016/s0092-8674(00)80447-x

[46] Wellems TE, Walker-Jonah A, Panton LJ: Genetic mapping of the chloroquine-resistance locus on *Plasmodium falciparum* chromosome 7. *Proc. Natl Acad. Sci. USA* 88(8) 3382–3386 (1991). 1673031 10.1073/pnas.88.8.3382 PMC51451

[47] Wünsch S, Sanchez CP, Gekle M, Grobe-Wortmann L, Wiesner J, Lanzer M: Differential stimulation of the Na^+^/H^+^ exchanger determines chloroquine uptake in *Plasmodium falciparum* *J. Cell Biol.* 140 335–345 (1998). 9442109 10.1083/jcb.140.2.335 PMC2132566

[48] Sanchez CP, Horrocks P, Lanzer M: Is the putative chloroquine resistance mediator cg2 the Na^+^/H^+^ exchanger of *Plasmodium falciparum* ? *Cell* 92 601–602 (1998). 9506514 10.1016/s0092-8674(00)81127-7

[49] Wellems TE, Wootton JC, Fujioka H *et al.*: *P. falciparum* CG2, linked to chloroquine resistance, does not resemble Na^+^/H^+^ exchangers. *Cell* 94 285–286 (1998). 9708730 10.1016/s0092-8674(00)81471-3

[50] Cooper RA, Papakrivos J, Lane KD, Fujioka H, Lingelbach K, Wellems TE: PfCG2, a *Plasmodium falciparum* protein peripherally associated with the parasitophorous vacuolar membrane, is expressed in the period of maximum hemoglobin uptake and digestion by trophozoites. *Mol. Biochem. Parasitol.* 144(2) 167–176 (2005). 16183150 10.1016/j.molbiopara.2005.07.009

[51] Bray PG, Janneh O, Raynes KJ, Mungthin M, Ginsburg H, Ward SA: Cellular uptake of chloroquine is dependent on binding to ferriprotoporphyrin IX and is independent of NHE activity in *Plasmodium falciparum* *J. Cell Biol.* 145 363–376 (1999). 10209030 10.1083/jcb.145.2.363 PMC2133118

[52] Bennett TN, Patel J, Ferdig MT, Roepe PD: Altered *Plasmodium falciparum* Na^+^/H^+^ exchange activity is correlated with quinine resistance. *Mol. Biochem. Parasitol.* 153 48–58 (2007). 17353059 10.1016/j.molbiopara.2007.01.018 PMC2747254

[53] Fidock DA, Nomura T, Cooper RA, Su X, Talley AK, Wellems TE: Allelic modifications of the *cg2* and *cg1* genes do not alter the chloroquine response of drug-resistant *Plasmodium falciparum* *Mol. Biochem. Parasitol.* 110(1) 1–10 (2000). 10989140 10.1016/s0166-6851(00)00249-8

[54] Martiney JA, Ferrer AS, Cerami A, Dzekunov S, Roepe PD: Chloroquine uptake, altered partitioning and the basis of drug resistance: evidence for a role of chloride-dependent ionic regulation. In: *Transport and trafficking in the malaria infected erythrocyte* Wiley, Winchester, UK, 265–281 (1999). 10.1002/9780470515730.ch18 10645551

[55] Fidock DA, Nomura T, Talley AK *et al.*: Mutations in the digestive vacuole transmembrane protein *pfcrt* and evidence for their role in chloroquine resistance. *Mol. Cell.* 6 861–871 (2000). 11090624 10.1016/s1097-2765(05)00077-8 PMC2944663

[56] Sidhu AB, Verdier-Pinard D, Fidock DA: Chloroquine resistance in *Plasmodium falciparum* malaria parasites conferred by *pfcrt* mutations. *Science* 298 210–213 (2002). 12364805 10.1126/science.1074045 PMC2954758

[57] Cooper RA, Ferdig MT, Su X *et al.*: Alternative mutations at position 76 of the vacuolar transmembrane protein PfCRT produce chloroquine resistance and unique stereospecific quinine and quinidine responses in *Plasmodium falciparum* *Mol. Pharmacol.* 61 1–8 (2002). 11752204 10.1124/mol.61.1.35

[58] Reed MB, Saliba KJ, Caruana SR, Kirk K, Cowman AF: Pgh1 modulates sensitivity and resistance to multiple antimalarials in *Plasmodium falciparum* *Nature* 403 906–909 (2000). 10706290 10.1038/35002615

[59] Sidhu AB, Valderramos SG, Fidock DA: *pfmdr1* mutations contribute to quinine resistance and enhance mefloquine and artemisinin sensitivity in *Plasmodium falciparum* *Mol. Microbiol.* 57(4) 913–926 (2005). 16091034 10.1111/j.1365-2958.2005.04729.x

[60] Ferdig MT, Cooper RA, Mu J *et al.*: Dissecting the loci of low-level quinine resistance in malaria parasites. *Mol. Microbiol.* 52(4) 985–997 (2004). 15130119 10.1111/j.1365-2958.2004.04035.x

[61] Krogstad DJ, Gluzman IY, Kyle DE *et al.*: Efflux of chloroquine from *Plasmodium falciparum*: mechanism of chloroquine resistance. *Science* 238 1283–1285 (1987). 3317830 10.1126/science.3317830

[62] Barnes DA, Foote SJ, Galatis D, Kemp DJ, Cowman AF: Selection for high-level chloroquine resistance results in deamplification of the *pfmdr1* gene and increased sensitivity to mefloquine in *Plasmodium falciparum* *EMBO J.* 11 3067–3075 (1992). 1353446 10.1002/j.1460-2075.1992.tb05378.x PMC556790

[63] Foote SJ, Kyle DE, Martin RK *et al.*: Several alleles of the multidrug-resistance gene are closely linked to chloroquine resistance in *Plasmodium falciparum* *Nature* 345 255–258 (1990). 2185424 10.1038/345255a0

[64] Mehlotra RV, Fujioka H, Roepe PD *et al.*: Evolution of new *Plasmodium falciparum* chloroquine resistance phenotype in association with *pfcrt*polymorphisms in Papua New Guinea and South America. *Proc. Natl Acad. Sci. USA* 98(22) 12689–12694 (2001). 11675500 10.1073/pnas.221440898 PMC60115

[65] Chen N, Kyle DE, Pasay C *et al.*: *Pfcrt* allelic types with two novel amino-acid mutations in chloroquine-resistant *Plasmodium falciparum* isolates from the Phillipines. *Antimicrob. Agents Chemother.* 47(11) 3500–3505 (2003). 14576108 10.1128/AAC.47.11.3500-3505.2003 PMC253797

[66] Lim P, Chy S, Ariey F *et al.*: Pfcrt polymorphism and chloroquine resistance in *Plasmodium falciparum* strains isolated in Cambodia. *Antimicrob. Agents Chemother.* 47(11) 87–94 (2003). 12499174 10.1128/AAC.47.1.87-94.2003 PMC149020

[67] Cowman AF, Galatis D, Thompson JK: Selection for mefloquine resistance in *Plasmodium falciparum* is linked to amplification of the *pfmdr1* gene and cross-resistance to halofantrine and quinine. *Proc. Natl Acad. Sci. USA* 91 1143–1147 (1994). 8302844 10.1073/pnas.91.3.1143 PMC521470

[68] Ritchie GY, Mungthin M, Green JE, Bray PG, Hawley SR, Ward SA: *In vitro* selection of halofantrine resistance in *Plasmodium falciparum* is not associated with increased expression of Pgh1. *Mol. Biochem. Parasitol.* 83 35–46 (1996). 9010840 10.1016/s0166-6851(96)02746-6

[69] Dorsey G, Kamya MR, Singh A, Rosenthal PJ: Polymorphisms in the *Plasmodium falciparum pfcrt* and *pfmdr1* genes and clinical response to chloroquine in Kampala, Uganda. *J. Infec. Dis.* 183 1417–1420 (2001). 11294677 10.1086/319865

[70] Price RN, Uhlemann AC, Brockman A *et al.*: Mefloquine resistance in *Plasmodium falciparum* and increased *pfmdr1* gene copy number. *Lancet* 364(9432) 438–447 (2004). 15288742 10.1016/S0140-6736(04)16767-6 PMC4337987

[71] Ursos L, Dzekunov S, Roepe PD: The effects of chloroquine and verapamil on digestive vacuolar pH of *P. falciparum* either sensitive or resistant to chloroquine. *Mol. Biochem. Parasitol.* 110 125–134 (2000). 10989150 10.1016/s0166-6851(00)00262-0

[72] Zhang H, Howard EM, Roepe PD: Analysis of the antimalarial drug resistance protein PfCRT expressed in yeast. *J. Biol. Chem.* 277 49767–49775 (2002). 12351620 10.1074/jbc.M204005200

[73] Zhang H, Paguio M, Roepe PD: The antimalarial drug resistance protein *Plasmodium falciparum* chloroquine resistance transporter binds chloroquine. *Biochemistry* 43(26) 8290–8296 (2004). 15222741 10.1021/bi049137i

[74] Naude B, Brzostowski JA, Kimmel AR, Wellems TE: Dictyostelium discoideum expresses a malaria chloroquine resistance mechanism upon transfection with mutant, but not wild-type, *Plasmodium falciparum* transporter PfCRT. *J. Biol. Chem.* 280(27) 25596–25603 (2005). 15883156 10.1074/jbc.M503227200 PMC1779819

[75] Johnson DJ, Fidock DA, Mungthin M *et al.*: Evidence for a central role for PfCRT in conferring *Plasmodium falciparum* resistance to diverse antimalarial agents. *Mol. Cell.* 15(6) 867–877 (2004). 15383277 10.1016/j.molcel.2004.09.012 PMC2943419

[76] Reeves DC, Liebelt DA, Lakshmanan V, Roepe PD, Fidock DA, Akabas MH: Chloroquine-resistant isoforms of the *Plasmodium falciparum* chloroquine resistance transporter acidify lysosomal pH in HEK293 cells more than chloroquine-sensitive isoforms. *Mol. Biochem. Parasitol.* 150(2) 288–299 (2006). 17014918 10.1016/j.molbiopara.2006.09.001 PMC1687154

[77] Bosia A, Ghigo D, Turrini F, Nissani E, Pescarmona GP, Ginsburg H: Kinetic characterization of Na^+^/H^+^ antiport of *Plasmodium falciparum* membrane. *J. Cell. Physiol.* 154(3) 527–534 (1993). 8382209 10.1002/jcp.1041540311

[78] Spillman NJ, Allen RJ, Kirk K: Acid extrusion from the intraerythrocytic malaria parasite is not via a Na^+^/H^+^ exchanger. *Mol. Biochem. Parasitol.* 162(1) 96–99 (2008). 18675853 10.1016/j.molbiopara.2008.07.001

[79] Roepe PD, Ferdig MT: *P. falciparum* Na^+^/H^+^ exchanger (PfNHE) function and quinine resistance (QNR). *Mol. Biochem. Parasitol.* 166 1–2 (2009). 19428665 10.1016/j.molbiopara.2009.01.008 PMC2768547

[80] Karcz SR, Herrmann VR, Trottein F, Cowman AF: Cloning and characterization of the vacuolar ATPase B subunit from *Plasmodium falciparum* *Mol. Biochem. Parasitol.* 65(1) 123–133 (1984). 10.1016/0166-6851(94)90121-x 7935619

[81] Hayward R, Saliba KJ, Kirk K: The pH of the digestive vacuole of *Plasmodium falciparum* is not associated with chloroquine resistance. *J. Cell Sci.* 119(Pt 6) 1016–1025 (2006). 16492710 10.1242/jcs.02795

[82] Dzekunov S, Ursos L, Roepe PD: Digestive vacuolar pH of intact intraerythrocytic *P. falciparum* either sensitive or resistant to chloroquine. *Mol. Biochem. Parasitol.* 110 107–124 (2000). 10989149 10.1016/s0166-6851(00)00261-9

[83] Nessler S, Friedrich O, Bakouh N *et al.*: Evidence for activation of endogenous transporters in *Xenopus laevis* oocytes expressing the *Plasmodium falciparum* chloroquine resistance transporter, PfCRT. *J. Biol. Chem.* 279(38) 39438–39446 (2004). 15258157 10.1074/jbc.M404671200

[84] Klonis N, Tan O, Jackson K, Goldberg D, Klemba M, Tilley L: Evaluation of pH during cytostomal endocytosis and vacuolar catabolism of haemoglobin in *Plasmodium falciparum* *Biochem. J.* 407(3) 343–354. (2007). 17696875 10.1042/BJ20070934 PMC2275073

[85] Pan W, Ravot E, Tolle R *et al.*: Vaccine candidate MSP-1 from *Plasmodium falciparum*: a redesigned 4917 bp polynucleotide enables synthesis and isolation of full-length protein from *Escherichia coli* and mammalian cells. *Nucleic Acids Res.* 27(4) 1094–1103 (1999). 9927744 10.1093/nar/27.4.1094 PMC148291

[86] Sanchez CP, McLean JE, Rohrbach P, Fidock DA, Stein WD, Lanzer M: Evidence for a *pfcrt*-associated chloroquine efflux system in the human malarial parasite *Plasmodium falciparum* *Biochemistry* 44(29) 9862–9870 (2005). 16026158 10.1021/bi050061f

[87] Sanchez CP, Rohrbach P, McLean JE, Fidock DA, Stein WD, Lanzer M: Differences in trans-stimulated chloroquine efflux kinetics are linked to PfCRT in *Plasmodium falciparum* *Mol. Microbiol.* 64(2) 407–420 (2007). 17493125 10.1111/j.1365-2958.2007.05664.x PMC2944662

[88] Amoah LE, Lekostaj JK, Roepe PD: Heterologous expression and ATPase activity of mutant versus wild type PfMDR1 protein. *Biochemistry* 46(20) 6060–6073 (2007). 17469853 10.1021/bi7002026

[89] Lekostaj JK, Amoah LE, Roepe PD: A single S1034C mutation confers altered drug sensitivity to PfMDR1 ATPase activity that is characteristic of the 7G8 isoform. *Mol. Biochem. Parasitol.* 157(1) 107–111 (2008). 18006157 10.1016/j.molbiopara.2007.09.008 PMC2211713

[90] Lekostaj JK, Natarajan JK, Paguio MF, Wolf C, Roepe PD: Photoaffinity labeling of the *Plasmodium falciparum* chloroquine resistance transporter with a novel perfluorophenylazido chloroquine. *Biochemistry* 47(39) 10394–10406 (2008). 18767816 10.1021/bi8010658 PMC2724749

[91] Ekoue-Kovi K, Yearick K, Iwaniuk DP *et al.*: Synthesis and antimalarial activity of new 4-amino-7-chloroquinolyl amides, sulfonamides, ureas and thioureas. *Bioorg. Med. Chem.* 17(1) 270–283 (2009). 19041248 10.1016/j.bmc.2008.11.009 PMC2647815

[92] Cabrera M, Paguio M, Natarajan JK, Wolf C, Roepe PD: Chloroquine transport in chloroquine resistant *Plasmodium falciparum*: influx and efflux kinetics using a fluorescent chloroquine probe. Presented at: *American Society of Tropical Medicine, Hygiene Annual Meeting*. Philadelphia, PA, USA, 4–8 November 2007.

[93] Certain LK, Briceño M, Kiara SM, Nzila AM, Watkins WM, Sibley CH: Characteristics of *Plasmodium falciparum* *dhfr* haplotypes that confer pyrimethamine resistance, Kilifi, Kenya, 1987–2006. *J. Infect. Dis.* 197(12) 1743–1751 (2008). 18513156 10.1086/588198 PMC2680814

[94] Natarajan JK, Alumasa JN, Yearick K *et al.*: 4-N-, 4-S-, and 4-O-chloroquine analogues: influence of side chain length and quinolyl nitrogen pKa on activity vs chloroquine resistant malaria. *J. Med. Chem.* 51(12) 3466–3479 (2008). 18512900 10.1021/jm701478a PMC2637136

[95] Yearick K, Ekoue-Kovi K, Iwaniuk DP *et al.*: Overcoming drug resistance to heme-targeted antimalarials by systematic side chain variation of 7-chloro-4-aminoquinolines *J. Med. Chem.* 51(7) 1995–1998 (2008). 18345611 10.1021/jm800106u PMC2581884

[96] Vivas L, Rattray L, Stewart L *et al.*: Antimalarial efficacy of pyronaridine and artesunate in combination *in vitro* and *in vivo* *Acta Trop.* 105(3) 222–228 (2008). 18279817 10.1016/j.actatropica.2007.12.005

[97] Muangnoicharoen S, Johnson DJ, Looareesuwan S, Krudsood S, Ward SA: Role of known molecular markers of resistance in the antimalarial potency of piperaquine and dihydroartemisinin *in vitro* *Antimicrob. Agents Chemother.* 53(4) 1362–1366 (2009). 19188388 10.1128/AAC.01656-08 PMC2663099

[98] Davis CB, Bambal R, Moorthy GS *et al.*: Comparative preclinical drug metabolism and pharmacokinetic evaluation of novel 4-aminoquinoline antimalarials. *J. Pharm. Sci.* 98(1) 362–377 (2009). 18563832 10.1002/jps.21469

[99] Kelly JX, Winter R, Peyton DH, Hinrichs DJ, Riscoe M: Optimization of xanthones for antimalarial activity: the 3,6-bis-w-diethylaminoalkoxyxanthone series. *Antimicrob. Agents Chemother.* 46(1) 144–150 (2002). 11751125 10.1128/AAC.46.1.144-150.2002 PMC126978

[100] Nkrumah LJ, Riegelhaupt PM, Moura P *et al.*: Probing the multifactorial basis of *Plasmodium falciparum* quinine resistance: evidence for a strain-specific contribution of the sodium–proton exchanger PfNHE. *Mol. Biochem. Parasitol.* 165 122–131 (2009). 19428659 10.1016/j.molbiopara.2009.01.011 PMC3082771

